# Do feature selection methods for selecting environmental covariables enhance genomic prediction accuracy?

**DOI:** 10.3389/fgene.2023.1209275

**Published:** 2023-07-24

**Authors:** Osval A. Montesinos-López, Leonardo Crespo-Herrera, Carolina Saint Pierre, Alison R. Bentley, Roberto de la Rosa-Santamaria, José Alejandro Ascencio-Laguna, Afolabi Agbona, Guillermo S. Gerard, Abelardo Montesinos-López, José Crossa

**Affiliations:** ^1^ Facultad de Telemática, Universidad de Colima, Colima, Mexico; ^2^ International Maize and Wheat Improvement Center (CIMMYT), El Battan, Mexico; ^3^ Colegio de Postgraduados, Campus Tabasco, Tabasco, Mexico; ^4^ Instituto Mexicano del Transporte, Querétaro, Mexico; ^5^ International Institute of Tropical Agriculture (IITA), Ibadan, Nigeria; ^6^ Molecular & Environmental Plant Sciences, Texas A&M University, College Station, TX, United States; ^7^ Centro Universitario de Ciencias Exactas e Ingenierías (CUCEI), Universidad de Guadalajara, Guadalajara, JA, Mexico; ^8^ Colegio de Postgraduados, Campus Montecillos, Montecillos, Mexico

**Keywords:** genomic prediction, feature selection, environmental covariables, genotype x environment interaction, genomic selection

## Abstract

Genomic selection (GS) is transforming plant and animal breeding, but its practical implementation for complex traits and multi-environmental trials remains challenging. To address this issue, this study investigates the integration of environmental information with genotypic information in GS. The study proposes the use of two feature selection methods (Pearson’s correlation and Boruta) for the integration of environmental information. Results indicate that the simple incorporation of environmental covariates may increase or decrease prediction accuracy depending on the case. However, optimal incorporation of environmental covariates using feature selection significantly improves prediction accuracy in four out of six datasets between 14.25% and 218.71% under a leave one environment out cross validation scenario in terms of Normalized Root Mean Squared Error, but not relevant gain was observed in terms of Pearson´s correlation. In two datasets where environmental covariates are unrelated to the response variable, feature selection is unable to enhance prediction accuracy. Therefore, the study provides empirical evidence supporting the use of feature selection to improve the prediction power of GS.

## Introduction

To meet the demands of the growing population worldwide, it is of paramount importance to increase food production. However, it is a complex task to reach a significant increase because of the deterioration of natural resources, lack of land for agriculture use, significant fluctuations in the climatic conditions, *etc.* For this reason, novel alternatives, such as genomic selection (GS) methodology proposed by Meuwissen et al. (2001) are required for genetic improvement to increase yield stability, yield productivity, disease resistance, and nutrition and the subsequent end-use quality of key crops like wheat, rice, maize, and others (Crespo-Herrera, et al., 2021).

GS is a transformative approach that employs high-density markers across the entire genome and operates under the assumption that at least one genetic marker is in linkage disequilibrium with a causative QTL for the desired trait (Meuwissen et al., 2001). This methodology is reshaping the field of plant and animal breeding through various means: (a) preemptively identifying promising genotypes prior to planting, (b) enhancing the accuracy of selecting superior individuals, (c) yielding significant resource savings primarily by reducing the need for extensive phenotyping, (d) accelerating the variety development process by reducing cycle length, (e) intensifying selection efforts, (f) facilitating the selection of traits that are challenging to measure, and (g) enhancing the accuracy of the selection process.

Most of the referenced advantages of the GS are because the genomic selection methodology is predictive (Meuwissen et al., 2001). For this reason, the GS methodology trains statistical machine learning models with a reference population that contains phenotypic and genotypic records, and after the training process, produces predictions for candidates individuals that only contain genotypic records (Desta and Ortiz, 2014). The term “statistical machine learning methods” refers to the methodologies that originate from the domains of statistics and machine learning, as defined by Montesinos-López et al. (2022).

GS methodology is being applied in breeding programs of many crops like groundnut, maize, cassava, wheat, rice, chickpea, *etc.* (Roorkiwal et al., 2016; Crossa et al., 2017; Wolfe et al., 2017; Huang et al., 2019). However, the practical implementation of GS is still challenging because GS depends on many factors that should be optimized to guarantee high prediction accuracies. However, the simultaneous optimization of all these factors is very complex and so unexpected results are often found.

Several factors influence the application of the GS methodology, including: (a) the specific prediction objectives pertaining to tested lines in untested environments, untested lines in untested environments, tested lines in tested environments, or untested lines in tested environments, (b) the selection of an appropriate statistical machine learning method for accurate predictions (Montesinos-López et al., 2022), (c) the extent of coverage and marker density employed, (d) the influence of population structure (Frouin et al., 2019), (e) the heritability of the trait being considered, (f) the degree of mismatch between individuals in the testing and training sets (Habier et al., 2007), and (g) the sample size of the testing and training sets.

The calibration process necessary for the effective implementation of the GS methodology is not a simple task. However, accumulating empirical evidence from both small- and large-scale breeding programs indicates the feasibility of utilizing this methodology successfully. Nevertheless, it is crucial to acknowledge that achieving success requires careful consideration of various factors influencing its accuracy. Failing to account for these factors may result in unexpected outcomes following implementation. Specifically, important details such as the computation method for best linear unbiased estimates (BLUEs) used as response variables and the trait heritability play a pivotal role in avoiding low prediction accuracies.

Ongoing research aims to enhance the accuracy of GS by optimizing various factors. One area of focus is training and testing optimization, which aims to improve the precision of this methodology (Rincent et al., 2012; Akdemir et al., 2015). Additionally, extensive evaluations of different statistical machine learning methods are underway to achieve better prediction performance and develop robust models that require minimal tuning while maintaining high accuracy (Montesinos-López et al., 2022). Furthermore, researchers are investigating the integration of other omics data (such as phenomics, transcriptomics, metabolomics, and Environics) to enhance the accuracy of GS and identify powerful predictors for the traits of interest (Montesinos-López et al., 2017; Krause et al., 2019; Monteverde et al., 2019; Costa-Neto et al., 2021a; Costa-Neto et al., 2021b; Hu et al., 2021; Wu et al., 2022). These efforts aim to leverage additional information sources to improve the predictive capabilities of the GS methodology.

Incorporating environmental information, also known as enviromic data, in genomic prediction models has yielded varying results. Some studies have reported significant improvements by including this information (Costa-Neto et al., 2021a; Costa-Neto et al., 2021b), while others have observed modest or no improvements (Monteverde et al., 2019; Jarquin et al., 2020; Rogers and Holland, 2022). These mixed findings highlight the absence of a robust and precise method for effectively integrating environmental information into genomic prediction models. To address this gap, our present study focuses on evaluating the use of feature selection to identify the optimal environmental predictors. The goal is to enhance the accuracy of the GS methodology when incorporating environmental covariates.

Feature selection involves choosing a subset of relevant features from a larger available dataset. It plays a vital role in statistical machine learning as selecting the appropriate features can significantly improve model accuracy and efficiency. By selecting only the most pertinent features, feature selection reduces model complexity, leading to faster training times, better generalization to new data, and more interpretable outcomes. Additionally, feature selection helps mitigate overfitting, which occurs when a model becomes excessively complex and memorizes the training data rather than learning general patterns applicable to new data. Overall, feature selection is a critical step in the statistical machine learning pipeline, with the potential to greatly impact the performance and interpretability of a model (Chandrashekar and Sahin, 2014).

In this research, two feature selection methods (Pearson´s correlation and Boruta) were evaluated, and their performance was compared without incorporating the environmental covariates to evaluate the increase of prediction accuracy that is reached by adding the selected environmental covariates with the two methods of feature selection.

## Materials and methods

A summary of the 6 datasets utilized in this study is in Table 1.

**TABLE 1:** Summary of the six datasets. Num. Lines denotes the number of lines evaluated in each data set. Num. Denotes number. USP denotes the University of São Pablo data set.

Dataset	Environments		Traits		Num. Lines
	Description	Num	Description	Num	
*USP*	Env1, Env2, Env3, Env4	4	GY	1	100
Indica	2010, 2011, 2012	3	GC, GY, PH, PHR	4	327
Japonica	2009, 2010, 2011, 2012, 2013	5	GC, GY, PH, PHR	4	320
G2F_2014	DEH1, IAH1ab, IAH1c, IAH2, IAH3, IAH4, ILH1, INH1, MNH1, MOH1, MOH2, NCH1, NEH1, NEH2, NEH3, NYH1, TXH2, WIH1	18	Grain_Moisture_BLUE, Grain_Moisture_weight, Yield_Mg_ha_BLUE, Yield_Mg_ha_weight	4	781
G2F_2015	DEH1, GAH1, INH1, MNH1, NCH1, NEH1_NEH4, NEH2, NEH3, NYH2, NYH3, OHH1, SDH1	12	Grain_Moisture_BLUE, Grain_Moisture_weight, Yield_Mg_ha_BLUE, Yield_Mg_ha_weight	4	1,011
G2F_2016	ARH1, DEH1, GAH1, IAH1, IAH2, IAH3, IAH4, ILH1, INH1, MIH1, MNH1, MOH1, NCH1, NEH1, NYH2, OHH1, WIH1, WI	18	Grain_Moisture_BLUE, Grain_Moisture_weight, Yield_Mg_ha_BLUE, Yield_Mg_ha_weight	4	456

### Data

#### Datasets 1. USP

The University of São Paulo (USP) dataset is derived from germplasm developed by the Luiz de Queiroz College of Agriculture at the University of São Paulo, Brazil. Between 2016 and 2017, an experiment was conducted using 49 inbred lines, resulting in 906 F1 hybrids, of which 570 were evaluated across eight different environments. The environments were created using a combination of two locations, 2 years, and two nitrogen levels. However, in this research, we used the information of 4 environments and 100 hybrids in each environment. The environments used in the study were distinct, with different soil types and climates. Environmental data was collected and used to create 248 covariables. The parent lines were genotyped using the Affymetrix Axiom Maize Genotyping Array, resulting in 54,113 high-quality SNPs after quality control procedures were applied. For more details about this data, see Costa-Neto, et al. (2021a).

#### Datasets 2. Indica

Indica (Monteverde et al., 2019) is a rice dataset comprising phenotypic information for four traits: Percentage of Head Rice Recovery (PHR measured in grams, as the weight of whole milled kernels, using a 100 g sample of rough rice), Grain Yield (GY of paddy rice in kilograms per hectare), Plant Height (PH measured in cm from the soil surface to the tip of the flag leaf) and percentage of Chalky Grain (GC measured as % of chalky kernels in a subsample of 50 g of total milled rice). These traits were measured in three developmental stages (maturation, reproductive, and vegetative) across three environments (2010, 2011, and 2012). In each environment were evaluated 327 genotypes and 18 environmental covariates were measured. These covariates include MaxTemp (maximum temperature in °C), MinTemp (minimum temperature in °C), TankEv (tank water evaporation in mm), Wind (wind speed in 2 m/km/24 h), PicheEv (pichi evaporation in mm), MinRelH (minimum relative humidity in %), AccumPpit (accumulated precipitation in mm), Sunhs (sunshine duration in hours), MinT15 (minimum temperature below 15°C in days), ThermAmp (thermal amplitude in °C), RelSun (relative sunshine duration in %), SolRad (solar radiation in cal/cm2/day), EfPpit (effective precipitation in mm), DegDay (degrees day in rice in °C), RelH (relative humidity in hours), PpitDay (precipitation day in days), MeanTemp (mean temperature in °C, Average of temperature over 24 h (0–24 h)), and AvTemp (average temperature in °C calculated as daily (Max + Min)/2). The dataset contains 981 assessments, with each line assessed once in each environment. For each line, 16,383 SNP markers were evaluated after quality control, with each marker coded as 0, 1, or 2.

#### Datasets 3. Japonica

Japonica is a dataset of 320 genotypes from the tropical rice Japonica population. It was evaluated for the same four traits (PH, PHR, GY, GC) as the indica population in five environments (2009–2013), with covariates measured three times in each year for the three developmental stages (maturation, reproductive, and vegetative). A total of 1,051 assessments were made in the five environments, and the dataset is not balanced. Additionally, each genotype was evaluated for 16,383 SNP markers that remained after quality control, with each marker coded as 0, 1, or 2. For more details about this data, see Monteverde, et al. (2019).

#### Datasets 4–6

The three datasets included in this study correspond to the years 2014 (data set 4), 2015 (data set 5), and 2016 (data set 6) of the Genomes to Fields maize project, as described by Rogers et al. (2021). These datasets comprise phenotypic, genotypic, and environmental information. Specifically, the phenotypic data utilized in this study focused on four specific traits, namely, Grain_Moisture_BLUE (GM_BLUE), Grain_Moisture_weight (GM_ Weight), Yield_Mg_ha_BLUE (YM_BLUE), and Yield_Mg_ha_weight (YM_Weight), selected from a larger set of traits reported by Rogers et al. (2021).

For years 2014 (data set 4), 2015 (data set 5) and 2016 (data set 6) there were 18, 12 and 18 available environments respectively (See Table 1). In terms of the number of genotypes evaluated for each year, there were 781, 1,011, and 456 genotypes for the years 2014 (data set 4), 2015 (data set 5), and 2016 (data set 6), respectively. The analysis utilized a set of 20,373 SNP markers that had already been imputed and filtered, as described by Rogers et al. (2021). The additive allele calls were recorded as counts of the minor allele (0, 1, 2). For further details regarding these datasets, please refer to Rogers et al. (2021).

There are unique values of environments for each dataset, while for traits, the G2F_2014, G2F_2015, and G2F_2016 datasets share the same traits, and the same happens with Indica and Japonica.

### Models

#### Bayesian model

The Bayesian model used with all predictors given in Table 2 is

$$Y_{ij}=\mu +E_{i}+g_{j}+gE_{ij}+\sum_{k=1}^{r}X_{ik}\beta_{k}+\epsilon_{ij}$$ (1)

**TABLE 2:** Description of the 15 predictors implemented. Environmental covariates (0 denotes no used, while 1 denotes used), selection method of environmental covariates (C= Pearson´s correlation and B=Boruta). TC denotes threshold correlation and this takes values of 0.3, 0.4, 0.5, 0.6, and 0.7. The largest TC value was evaluated first and in case that any covariate satisfied this TC value was used the second largest and so on.

Model	Predictor	Environmental covariates	Selection method	Average of covariates	Correlation
M0	**K.e** + **K**.g + **K**.**ge**	0	-	0	-
M1	**K**.e + **K**.g + **K**.ge+ $X_{e}$	1	-	0	>0
M2	**K**.ec + **K**.g + **K**.gec	1	C	0	TC
M3	**K**.e + **K.**g + **K**.ge+ $X_{e}$	1	C	0	TC
M4	**K**.ec + **K**.g + **K**.gec+ $X_{e}$	1	C	0	TC
M5	**K**.e + **K**.g + **K**.ge+ $X_{e2}$	1	C	0	TC
M6	**K**.e + **K**.g + **K**.ge+ $X_{e.avg}$	1	C	1	TC
M7	**K**.ec + **K**.g + **K**.gec+ $X_{e.avg}$	1	C	1	TC
M8	**K**.ec + **K**.g + **K**.gec+ $X_{e.avg}$	1	C and B	1	TC
M9	**K**.e + **K**.ge + **X**.g.ec	1	B	0	-
M10	**K**.e + **K**.g + **K**.ge+ $X_{e}$ (Tenative true)	1	B	0	-
M11	**K**.ec + **K**.g + **K**.gec+ $X_{e.avg}$ (Tentative false)	1	B	1	-
M12	**K**.ec + **K**.g + **K**.ge+ $X_{e.avg}$ (Tenative true)	1	B	1	-
M13	**K**.ec + **K**.g + **K**.gec+ $X_{e.avg}$ (Tenative true)	1	B	1	-
M14	**K**.ec + **K**.g + **K**.gec+ $X_{e.avg}$ (Tenative False)	1	B	1	-

Where $Y_{ij}$ is the response variable for the genotype j in environment i, μ is a general mean, $E_{i}$ are the random effects of locations (environments) distributed as $E={\left(E_{1},\ldots ,E_{I}\right)}^{T}∼N\left(0,\sigma_{E}^{2}H.e\right)$ , where H.e is the environmental relationship matrix as computed by Cuevas et al. (2016), but in place of using genomic information, it was computed using environmental variables; that is, $H.e=\frac{X_{e}X_{e}^{T}}{r}$ , where $X_{e}=\left(X_{1},\ldots ,X_{r}\right)$ is the standardize (centered and scaled) matrix of dimension I×r containing the environmental information of I environments and for each environment were measured r environmental covariates; $X_{ik}$ denotes the environmental covariate k measured in environment i, $\beta_{k}$ is the beta coefficient corresponding to covariate $X_{ik}$ ; $g_{j},j=1,\ldots ,J$ , are the random effects of genotypes (lines), $gE_{ij}$ are the random effects of genotype × environment interaction (GE) and $\epsilon_{ij}$ are the random error components in the model assumed to be independent normal random variables with mean 0 and variance $\sigma^{2}$ . Furthermore, it is assumed that $g={\left(g_{1},\ldots ,g_{J}\right)}^{T}∼N\left(0,\sigma_{g}^{2}K.g\right)$ , where K.g is the genomic relationship matrix as computed by (Cuevas et al., 2016; slightly different as proposed by VanRaden (2008), using the marker data ( $K.g=\frac{M_{e}M_{e}^{T}}{p}$ ) where $M_{e}$ is the standardize (centered and scaled) matrix of dimension J×p containing the marker information of J genotypes for which were measured p markers. $gE={\left(gE_{11},\ldots ,gE_{1J},\ldots ,gE_{IJ}\right)}^{T}∼N\left(0,{K.gec\sigma }_{gE}^{2}\right)$ , where $K.gec=K.ec⊙Z_{g}K.g\;Z_{g}^{T}$ , where $K.ec=Z_{e}H.e\;Z_{e,}^{T}\;Z_{e}$ is the design matrix of environments, ⊙ denotes the Hadamard product and $Z_{g}$ is the design matrix of genotypes. It is important to point out that the dimension of $X_{e}$ is reduced after variable selection and in place of being I×r is $I\times r_{s}$ with $r_{s}\leq r$ .

#### Predictors implemented

To understand better the content of Table 2, next we describe how were computed some components of the predictors given in Table 2. For example, K.e was computed as $K.e=\frac{Z_{e}Z_{e}^{T}}{I}$ , $K.ge=K.e⊙Z_{g}K.g\;Z_{g}^{T}$ , $X_{e.avg}$ denotes an average covariate that was computed with the environmental covariates $\left(X_{e}\right),X_{e}$ use all the available environmental covariates when not was applied feature selection and only those selected with the feature selection methods when feature selection was applied. This average covariate ( $X_{e.avg}$ ) was computed from $X_{e}$ of order $I\times r_{s}$ after variable selection as:

Step 1. First, we identify the direction (positive and negative) of the correlation of each column of $X_{e}$ computed only with the response variable of the training set.

Step 2. Those columns of $X_{e}$ , with negative correlation are multiply by −1 to guarantee a positive correlation with the response variable and call these new matrix as $X_{e}^{*}$ .

Step 3. Then we compute $X_{e.avg}$ for the whole data set as the average of each row of $X_{e}^{*}$ and for this reason $X_{e.avg}$ has order I×1 . But because the covariates are measured at environment (Location) level to be in agreement with all design matrices given in model 1), $X_{e.avg}$ is expanded to order IJ×1 , since each covariate is the same for all lines in the same environment.

Using only one covariate as $X_{e.avg},$ implies that only one beta coefficient needs to be estimated in place of $r_{s}$ beta coefficients required when is used as input the $X_{e}$ matrix. In predictor M9 it is important to point out that **X**.g.ec denotes the covariates selected, but in place of selecting only from the environmental covariates we performed the Boruta selection from the markers and environmental covariates together. For feature selection the Pearson´s correlation and Boruta method were used, which are explained in the next section. All predictors given in Table 2 were implemented in the BGLR package of Pérez and de los Campos (2014) in the R statistical software (R Core Team, 2023).

### Feature selection methods and algorithms for selecting environmental covariates

The training of each model differs in the use of the environmental covariates that make up each dataset. Therefore, model M0 differs from the other models by making predictions without including any information given by the environmental covariates. For this reason, in model M0 the linear kernels $K.e=\frac{Z_{e}Z_{e}^{T}}{I}$ , $K.ge=K.e⊙Z_{g}K.g\;Z_{g}^{T}$ , were computed only with the design matrices of environments ( $Z_{e}$ ). On the other hand, model M1 is equal to model M0 plus adding all available environmental information as covariates $\left(X_{e}\right)$ without variable selection. Model M2 is equal to model M0, but with the difference that the computation of the linear kernels ( $K.ec=Z_{e}H.e\;Z_{e}^{T}$ and $K.gec=K.ec⊙Z_{g}K.g\;Z_{g}^{T}$ ) take into account the environmental covariates after variable selection with Pearson correlation. Model M3 is equal to model M1 but using the covariates, $X_{e},$ after variable selection with Pearson´s correlation. Model M4 is equal to model M2 but also including the environmental information as covariates ( $X_{e}$ ) after variable selection with Pearson´s correlation. Model M5 is equal to model M3 but in place of using only $X_{e},$ as covariates, after variable selection with Pearson´s correlation, also were used the square of each column of $X_{e},$ as covariates, that is as covariates used were $X_{e2}=X_{e}+X_{e}*X_{e}$ . Model M6 is equal to model M3 but in place of using $X_{e}$ , after variable selection with Perason´s correlation as covariate used only the average covariate ( $X_{e.avg}$ ). Model M7 is equal to model M4 but in place of using $X_{e}$ , after variable selection with Perason´s correlation as covariate used also the average covariate ( $X_{e.avg}$ ). Model M8 is equal to model M7 but the variable selection process was done with both Pearson´s correlation and Boruta simultaneously. Model M9 performed variable selection of markers and environmental covariates simultaneously using the Boruta algorithm and the resulting selected covariates are called **X**.g.ec, while **K**.e and **K**.ge were computed only with the design matrix of environment ( $Z_{e}$ ). Model M10 is equal to model M3 but the selection of the environmental covariates was done with the Boruta algorithm selecting tentative and confirmative covariates. It is important to point out than in model M10 also the Boruta algorithm was applied to select the markers and then with the selected markers were computed the linear kernels of lines (**K**.g) and genotype by environment (**K**.ge) interaction. Model M11 is equal to model M8 but now the environmental and markers covariates were selected with the Boruta algorithm selecting only confirmative covariates. Model M12 is equal to model M11 but with the difference that with the Boruta algorithm were selected tentative and confirmative covariates. Model M13 is equal to model M12 but with the difference that the selected environmental covariates were also used to compute the linear kernels of environments (**K**.ec) and genotype by environment (**K**.gec) interaction. Finally model M14 is equal to model M13 but with the difference that with the Boruta algorithm were selected only confirmed features. More details of each predictor are given in Table 2.

It is important to point out that only model M1, used all environmental covariates, while the remaining models use a selected subset of all available environmental covariates. The choice of feature selection method to be used depends on the specific problem and the characteristics of the data. It is often necessary to try different methods and evaluate their performance to find the most suitable one for a particular task.

The first feature selection method corresponds to the correlation present between the environmental covariates and the response variable, where the selection is made according to the largest correlation present in each training set of each trait. However, it is important to note that the selection of these covariates is done discarding the response variables in the testing set (a complete environment in this case since we will implement only a leave one environment out cross-validation), that is, the environment that will be predicted. The threshold correlations to select environmental covariates were: 0.3, 0.4, 0.5, 0.6, and 0.7. When the correlations are lower than the 0.3 value, it implies the training process was done without any environmental covariates, however it is important to realize if only few covariates satisfied the threshold correlation of 0.7 only these covariates were used in the training process, but if any satisfied this threshold were used those that satisfied lower threshold (0.5), and so on.

The second feature selection method consists of implementing the Boruta algorithm, which seeks to capture covariates that are strongly or weakly relevant to the response variable. Also, the selection of the covariates to include in the training process of the models were obtained using the response variables corresponding only to the training set; the observations that will be part of the testing are not used for selecting the important environmental covariates.

Boruta is a feature selection algorithm that was designed to handle high-dimensional datasets with noisy features (Kursa and Rudnicki, 2010) The algorithm works by creating a shadow feature set, which is a copy of the original feature set with random permutations added. The shadow features are used as a control to determine whether a feature is statistically significant or not. The original features are considered relevant if their importance scores are significantly higher than the importance scores of their shadow features. Boruta is useful for datasets with a large number of noisy features, where traditional feature selection methods may struggle. However, it can be computationally expensive and may require careful parameter tuning to achieve optimal results (Kursa and Rudnicki, 2010).

The Boruta algorithm works in the following steps.

1. Create a shadow feature set by randomly permuting the values of each feature.
2. Train a random forest model on the original and shadow feature sets.
3. Calculate the feature importance scores for each original feature by comparing them to the importance scores of their shadow features.
4. Determine the maximum importance score for each feature.
5. Check whether the maximum importance score is statistically significant using the Binomial test. If it is significant, mark the feature as important. Otherwise, mark the feature as unimportant. The Binomial test is a statistical test used in Boruta to evaluate the significance of feature importance scores. It compares the observed number of successes (e.g., the number of times a feature’s importance score exceeds a threshold) with the expected number of successes under a null hypothesis. The test assesses whether the observed results are statistically significant or can be attributed to chance. In Boruta, the Binomial test is applied to determine if the feature importance scores are significantly higher than the importance scores of shadow features, indicating the importance of the original features (Kursa and Rudnicki, 2010).
6. Repeat steps 1–5 for a predetermined number of iterations.)
7. Rank the features by their importance scores and select the top n features for the final feature set. The Boruta algorithm selects the important features based on their labels. “Confirmed” features are considered important, while “Rejected” features are deemed unimportant. “Tentative” features can be considered less important or require further investigation.

### Evaluation of prediction accuracy

To assess the prediction accuracy, a leave one environment out (LOEO) cross-validation approach was employed for each dataset. This involved iteratively constructing the training set by excluding one environment (I-1 environments) while utilizing the remaining environment as the testing set. The evaluation methodology follows the approach described by Montesinos-López et al. (2022). However, as pointed out before since some of the predictors provided in Table 2 selected environmental covariates, the selection of these covariates was done after splitting the training and testing the data, and only the training was used for selection of the important covariates, because when using the whole data before splitting the data in training and testing the results are too optimistic due to a leakage of information. Data leakage, a significant issue in machine learning, happens when the data used to train an ML algorithm includes information the model is attempting to predict. The leakage of information is a primary error in machine learning, which can significantly impact the production performance and validation accuracy of the model since we obtain very optimistic results that are not translated for real applications.

The prediction accuracy was reported in terms of the Normalized Root Mean Squared Error (NRMSE) and Pearson´s correlation (Cor). Also, we computed the absolute value of the intercept (**b0**) and the absolute value of **b** = 1-slope by regressing the observed values *versus* the predicted values to inform about the quality of the Pearson´s correlation. In terms of Pearson´s correlation the closet to 1 the value the better the predictions, while in terms of NRMSE, **b0** and **b**, the closest to zero the better the predictions. Furthermore, we conducted a computation to determine the count of instances where model *m* outperformed model *m´* in terms of NRMSE (Normalized Root Mean Squared Error), considering *m* = 0, … , 14 and *m´ =* 0, … ,14, but *m* different of *m´*. This count was performed for each dataset, taking into consideration the specific traits and environments under evaluation. Also, we computed the Relative Efficiency (RE), in terms of NRMSE of each model regarding the worst model with the following expression:

$$RE=\left(\frac{NRMSE\left(M_{0}\right)}{NRMSE\left(M_{k}\right)}-1\right)\times 100$$ (2)

Where $M_{0}$ denotes model M0 without environmental covariates, with k=1,…,14 in each data set. While the relative efficiencies for Cor were computed using the values of Cor, with the following equation (Eq. 3):

$$RE=\left(\frac{Cor\left(M_{k}\right)}{Cor\left(M_{0}\right)}-1\right)\times 100$$ (3)

Also, RE were computed for **b0** and **b**, using Eq. 2, but in place of using NRMSE we used the values of **b0** and **b.**

## Results

The results are provided in sections for each data set.

### USP data

**FIGURE 1:**
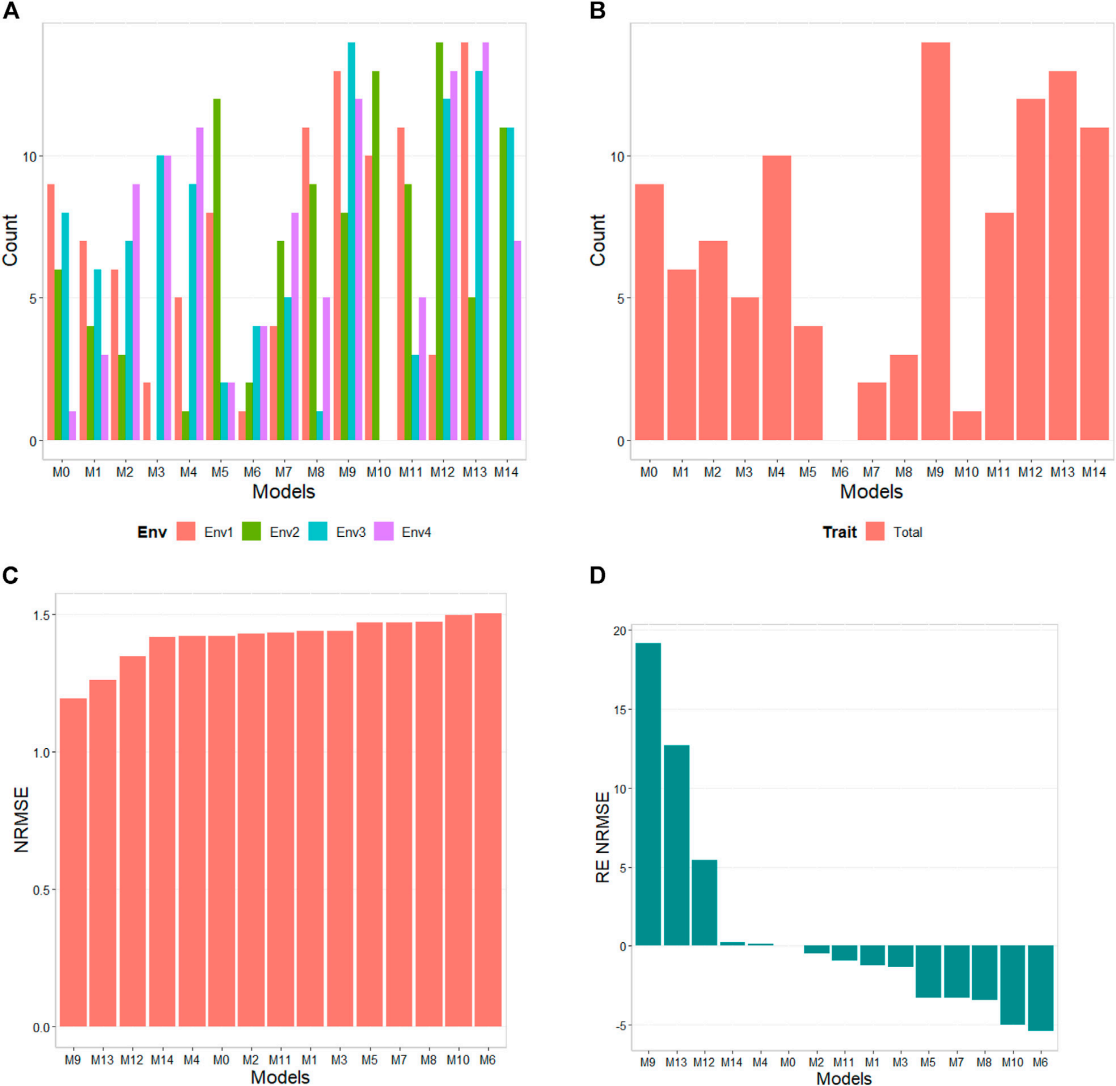
USP data. **(A)** Count of the number of times a model is better than another, by environment. **(B)** Count of the number of times a model is better than another, by trait. **(C)** Performance of the models’ prediction measured by Normalized Root Mean Squared Error (NRMSE). **(D)** Relative efficiency (RE) of each predictor compared to model M0, the model without environmental information.

The results of this dataset are presented in Figure 1 with details in Table 3. In the count by environments, the model M9 (47/60) turns out to be the best, with a count of 47 times it is better than the different models of a total combination of 60. The second-best model is M13 (46/60), while the worst models are M6 and M1 with counts of 11/60 and 20/60, respectively. Therefore, it is possible to observe a marked difference between the best and worst models, while the M0 model turns out to be the seventh-worst model. The M9 model (14/15) is observed as the best model with the maximum count (14 out of 14 possible combinations). The M13 model (13/14) is positioned as the second-best model. Meanwhile, the worst model turns out to be M6 (0/14), and the second-worst model is M10 (1/14). Thus, the results by environments and traits consistently point to the M9 model as the best and M6 as the model with the most deficient prediction. Furthermore, the M0 model appears as the fourth-worst model by environments and the fifth-worst model through the trait.

**TABLE 3:** University of São Paulo (USP) dataset. Count of the number of times a model was better than another in terms of Normalized Root Mean Squared Error (NRMSE), both by environments and by traits. Prediction accuracy in terms of NRMSE. b0 denotes the absolute values of the intercept and b denotes the absolute value of 1-slope. Under an ideal model both b0 and b should be equal to zero. Relative efficiency (RE) or each model in percentage was computed regarding model M0 without environmental covariates. When the percentage is positive there is a gain in prediction accuracy regarding M0, while when the percentage is negative there is a loss in terms of prediction accuracy of any model regarding M0.

Model	Env		Trait		NRMSE	NRMSE	Cor	Cor	b0	b0	b	b
	Won	%	Won	%		RE (%)		RE (%)		RE (%)		RE (%)
	Models		Models									
M0	24	40.00	9	64.29	1.420	0.00	0.427	0.00	1.527	0.00	0.326	0.00
M1	20	33.33	6	42.86	1.438	−1.25	0.424	−0.73	2.198	−30.54	0.273	19.23
M2	25	41.67	7	50.00	1.427	−0.50	0.448	4.97	0.318	380.31	0.079	310.98
M3	22	36.67	5	35.71	1.439	−1.34	0.434	1.69	2.788	−45.24	0.336	−3.18
M4	26	43.33	10	71.43	1.418	0.11	0.452	6.05	0.155	884.46	0.054	500.55
M5	24	40.00	4	28.57	1.468	−3.27	0.424	−0.66	1.841	−17.07	0.259	25.92
M6	11	18.33	0	0.00	1.501	−5.41	0.430	0.89	2.868	−46.75	0.365	−10.85
M7	24	40.00	2	14.29	1.468	−3.28	0.451	5.63	0.194	686.66	0.059	454.51
M8	26	43.33	3	21.43	1.470	−3.42	0.429	0.52	1.283	19.05	0.242	34.28
M9	47	78.33	14	100.00	1.192	19.13	0.206	−51.80	32.520	−95.30	5.559	−94.14
M10	23	38.33	1	7.14	1.494	−4.99	0.401	−6.09	2.670	−42.81	0.376	−13.52
M11	28	46.67	8	57.14	1.433	−0.92	0.429	0.49	1.237	23.42	0.243	34.12
M12	42	70.00	12	85.71	1.347	5.42	0.411	−3.77	0.180	747.34	0.035	838.04
M13	46	76.67	13	92.86	1.260	12.67	0.419	−1.90	1.008	51.42	0.237	37.57
M14	29	48.33	11	78.57	1.417	0.20	0.438	2.58	1.904	−19.81	0.421	−22.74

According to NRMSE, the M9 model is the best of all with a NRMSE = 1.19, the second-best model is M13 (NRMSE = 1.26). Meanwhile, the worst model is M6 (NRMSE = 1.50), followed by the second-worst model, M10 (NRMSE = 1.49). In terms of NRMSE the RE, compared to model (M0), the gains obtained are up to 19.13% for the best model, M9, but in terms of Cor, model M9 was worst than model M0 by 51.80%. However, in terms of Cor the best model was M4 and outperformed M0 by 6.05%. But it is interesting that model M4 improved regarding M0 in terms of the intercept (**b0**) and **b** by 884.46% and 500.55% respectively. See further details in Table 3.

### Indica data

**FIGURE 2:**
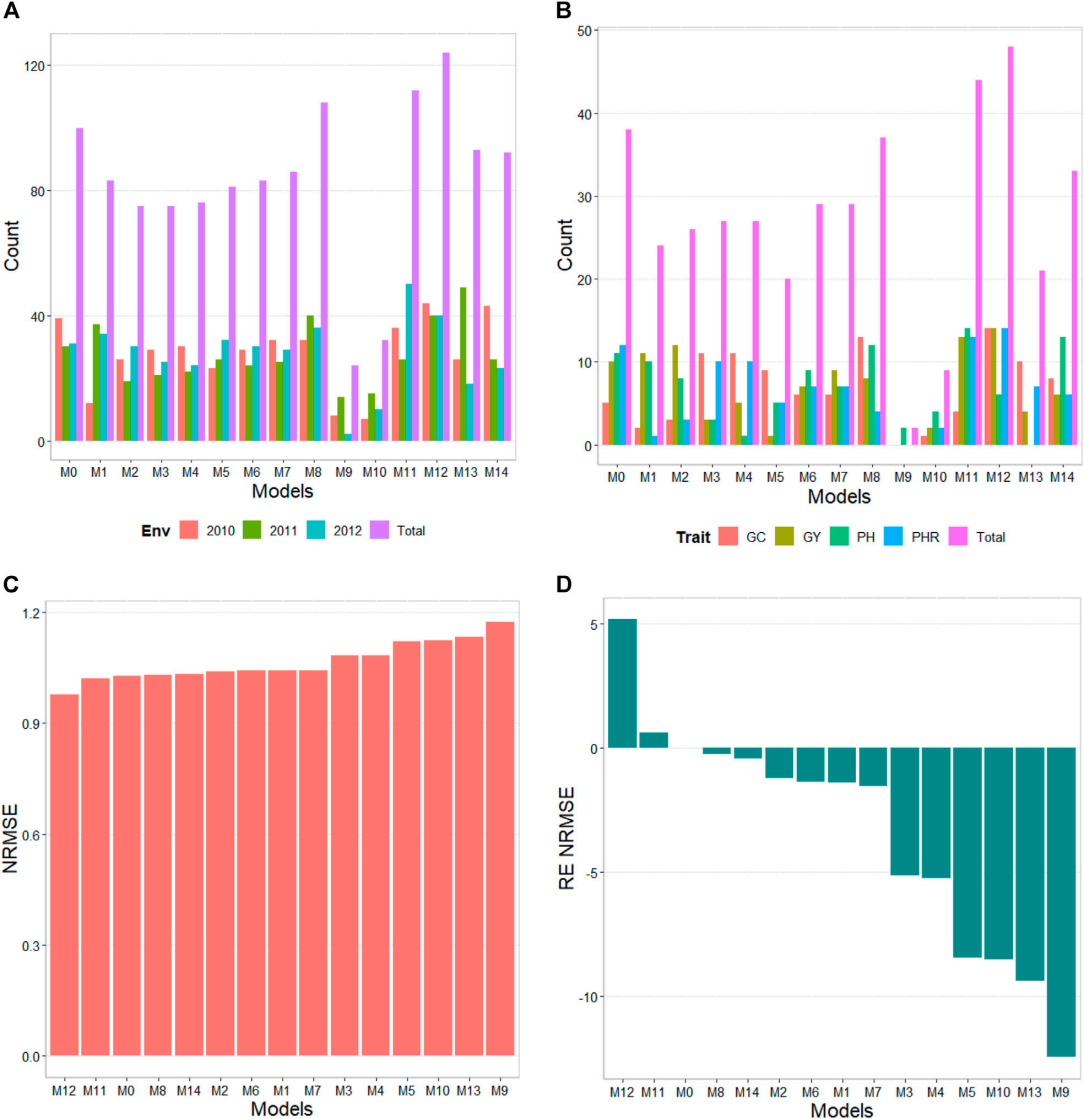
Indica data. **(A)** Count of the number of times a model is better than another, by environments. **(B)** Count of the number of times a model is better than another, by trait. **(C)** Prediction performance of each predictor in terms of Normalized Root Mean Squared Error (NRMSE). **(D)** Relative efficiency (RE) of each model compared to model M0, the model without environmental information.

The results of this dataset are presented in Figure 2 with details in Table 4. In the count by environments, model M12 turns out to be the best, winning in 124 out of 180 possible combinations (124/180). The second-best model is M11 (112/180), while the worst models are M9, M10, and M3 (with counts of 24/180, 32/180, and 75/180, respectively). Meanwhile, by trait, model M12 (48/60) is observed as the best model, model M11 (44/60) is positioned as the second-best model. In the opposite direction, the worst model is M9 (2/60) and the second-worst model is M10 (15/60). Thus, the results by environments and traits consistently point to model M12 as the best and M9 as the model with the most deficient prediction, highlighting model M0 among the top 5.

**TABLE 4:** Indica data. Count of the number of times a model was better than another in terms of Normalized Root Mean Squared Error (NRMSE), both by environments and by traits. Prediction accuracy in terms of NRMSE. b0 denotes the absolute values of the intercept and b denotes the absolute value of 1-slope. Under an ideal model both b0 and b should be equal to zero. Relative efficiency (RE) or each model in percentage was computed regarding model M0 without environmental covariates. When the percentage is positive there is a gain in prediction accuracy regarding M0, while when the percentage is negative there is a loss in terms of prediction accuracy of any model regarding M0.

Model	Env		Trait		NRMSE	NRMSE	Cor	Cor	b0	b0	b	b
	Won	%	Won	%		RE (%)		RE (%)		RE (%)		RE (%)
	Models		Models									
M0	100	55.56	38	63.33	1.027	0.00	0.511	0.00	3,023.024	0.00	0.364	0.00
M1	83	46.11	24	40.00	1.041	−1.40	0.513	0.22	2,993.455	0.99	0.375	−2.99
M2	75	41.67	26	43.33	1.040	−1.25	0.510	−0.25	3,012.109	0.36	0.432	−15.89
M3	75	41.67	27	45.00	1.082	−5.16	0.514	0.41	2,754.294	9.76	0.333	9.29
M4	76	42.22	27	45.00	1.084	−5.26	0.512	0.19	2,731.500	10.67	0.372	−2.25
M5	81	45.00	20	33.33	1.121	−8.46	0.512	0.13	2,661.258	13.59	0.327	11.19
M6	83	46.11	29	48.33	1.041	−1.37	0.514	0.44	2,548.352	18.63	0.293	24.07
M7	86	47.78	29	48.33	1.043	−1.56	0.512	0.18	2,634.676	14.74	0.339	7.30
M8	108	60.00	37	61.67	1.029	−0.27	0.513	0.22	2,794.690	8.17	0.299	21.45
M9	24	13.33	2	3.33	1.173	−12.46	0.072	−85.98	12,217.216	−75.26	2.047	−82.24
M10	32	17.78	9	15.00	1.122	−8.52	0.462	−9.61	7,927.607	−61.87	1.039	−65.01
M11	112	62.22	44	73.33	1.021	0.59	0.512	0.10	3,045.173	−0.73	0.330	10.13
M12	124	68.89	48	80.00	0.976	5.15	0.513	0.27	2,464.826	22.65	0.253	43.84
M13	93	51.67	21	35.00	1.13	−9.38	0.513	0.29	3,252.671	−7.06	0.306	18.62
M14	92	51.11	33	55.00	1.03	−0.44	0.512	0.02	2,780.146	8.74	0.348	4.48

By NRMSE, model M12 is the best with a NRMSE = 0.98 and the second-best model is M11 (NRMSE = 1.02). Meanwhile, the worst model is M9 (NRMSE = 1.17) and the second best is model M3 (RE = 1.13). In terms of NRMSE the RE of M12 compared to model (M0), showed a gain of only 5.15%, but in terms of Cor, model M12 only gain by 0.27%. However, in terms of Cor the best model was M6 and outperformed M0 by only 0.44%. Model M12 improved regarding M0 in terms of the intercept (**b0**) and **b** by 22.65% and 43.85% respectively (see Table 4 for more details).

Model M12 uses the Boruta method as the variable selection method. In this data set it is very interesting that model M0 without environmental covariates appears as one of the best, indicating for this dataset, that not necessary adding the environmental covariates is helpful to increase the prediction accuracy. One of the main reasons for this result is the low correlations of the environmental covariates with the response variable, causing them to add noise to the predictions when included.

### Japonica data

**FIGURE 3:**
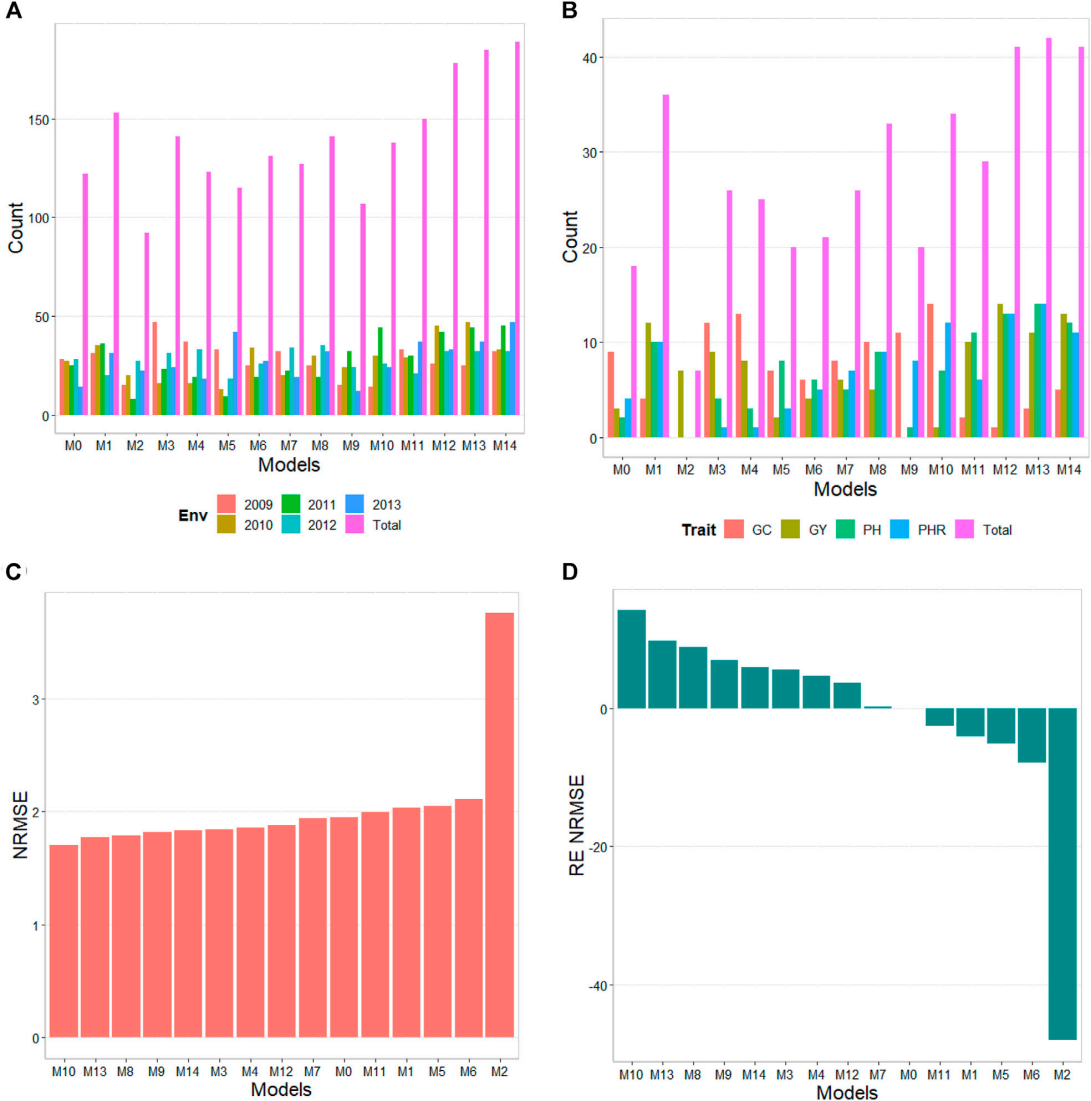
Japonica data. **(A)** Count of the number of times a model is found to be better than another, by environment. **(B)** Count of the number of times a model is found to be better than another, by trait. **(C)** Prediction accuracy of each predictor (M0 to M14) in terms of Normalized Root Mean Squared Error (NRMSE). **(D)** Relative efficiency (RE) of each model compared to model M0, the model without environmental information.

The results of this dataset are presented in Figure 3 (with details in Table 5). In the count by environments, model M14 turns out to be the best, winning in 189 out of 300 (189/300) possible combinations. The second-best model is M13 (185/300), while the worst models are M2 and M9, and M5 (with a count of 92/300 and 107/300, respectively). Meanwhile, by trait, model M13 (42/60) is observed as the best model, while models M14 (41/60) and M12 (41/60) are positioned as the second-best models. In the opposite direction, the worst model turns out to be M2 (7/60) and the second-worst model is M0 (18/60). Thus, the results by environments and traits are consistent in indicating model M13 as the best and M2 as the model with the poorest prediction, and model M0 is among the worst five models.

**TABLE 5:** Japonica data. Count of the number of times a model was better than another in terms of Normalized Root Mean Squared Error (NRMSE), both by environments and by traits. Prediction accuracy in terms of NRMSE. b0 denotes the absolute values of the intercept and b denotes the absolute value of 1-slope. Under an ideal model both b0 and b should be equal to zero. Relative efficiency (RE) or each model in percentage was computed regarding model M0 without environmental covariates. When the percentage is positive there is a gain in prediction accuracy regarding M0, while when the percentage is negative there is a loss in terms of prediction accuracy of any model regarding M0.

Model	Env		Trait		NRMSE	NRMSE	Cor	Cor	b0	b0	b	B
	Won	%	Won	%		RE (%)		RE (%)		RE (%)		RE (%)
	Models		Models									
M0	122	40.67	18	30.00	1.946	0.00	0.584	0.00	102.216	0.00	0.062	0.00
M1	153	51.00	36	60.00	2.029	−4.05	0.585	0.16	183.728	−44.37	0.069	−9.13
M2	92	30.67	7	11.67	3.752	−48.13	0.553	−5.27	151.100	−32.35	0.061	2.09
M3	141	47.00	26	43.33	1.843	5.62	0.584	−0.03	117.624	−13.10	0.067	−6.51
M4	123	41.00	25	41.67	1.858	4.78	0.523	−10.42	90.644	12.77	0.173	−63.86
M5	115	38.33	20	33.33	2.049	−5.00	0.587	0.51	25.585	299.51	0.084	−26.04
M6	131	43.67	21	35.00	2.111	−7.80	0.583	−0.19	46.789	118.46	0.069	−9.66
M7	127	42.33	26	43.33	1.940	0.35	0.526	−9.96	141.541	−27.78	0.147	−57.40
M8	141	47.00	33	55.00	1.786	8.97	0.536	−8.27	344.518	−70.33	0.100	−37.85
M9	107	35.67	20	33.33	1.818	7.04	0.020	−96.49	1793.721	−94.30	1.185	−94.73
M10	138	46.00	34	56.67	1.704	14.25	0.390	−33.19	970.116	−89.46	0.944	−93.39
M11	150	50.00	29	48.33	1.997	−2.51	0.567	−3.02	192.060	−46.78	0.001	4,205.17
M12	178	59.33	41	68.33	1.877	3.70	0.561	−3.91	24.735	313.24	0.061	2.97
M13	185	61.67	42	70.00	1.772	9.84	0.583	−0.14	130.979	−21.96	0.067	−6.44
M14	189	63.00	41	68.33	1.837	5.98	0.584	−0.03	126.899	−19.45	0.067	−7.52

In terms of NRMSE, model M10 is ranked the best with a NRMSE = 1.70, followed by the second-best model, M13 (NRMSE = 1.77). Meanwhile, the worst model is M2 (NRMSE = 3.75), and the second-worst model is M6 (NRMSE = 2.11). The gain of model M10 regarding model M0 in terms of NRMSE was of 14.25%, but this model reduced its accuracy in terms of Cor by 33.19%. While in terms of Cor the best model was M5 and outperformed M0 by only 0.51%. Model M5 improved regarding M0 in terms of the intercept (**b0**) by 299.51%, but was worst in terms of and **b** by 26.04% (Table 5).

### G2F_2014 data

**FIGURE 4:**
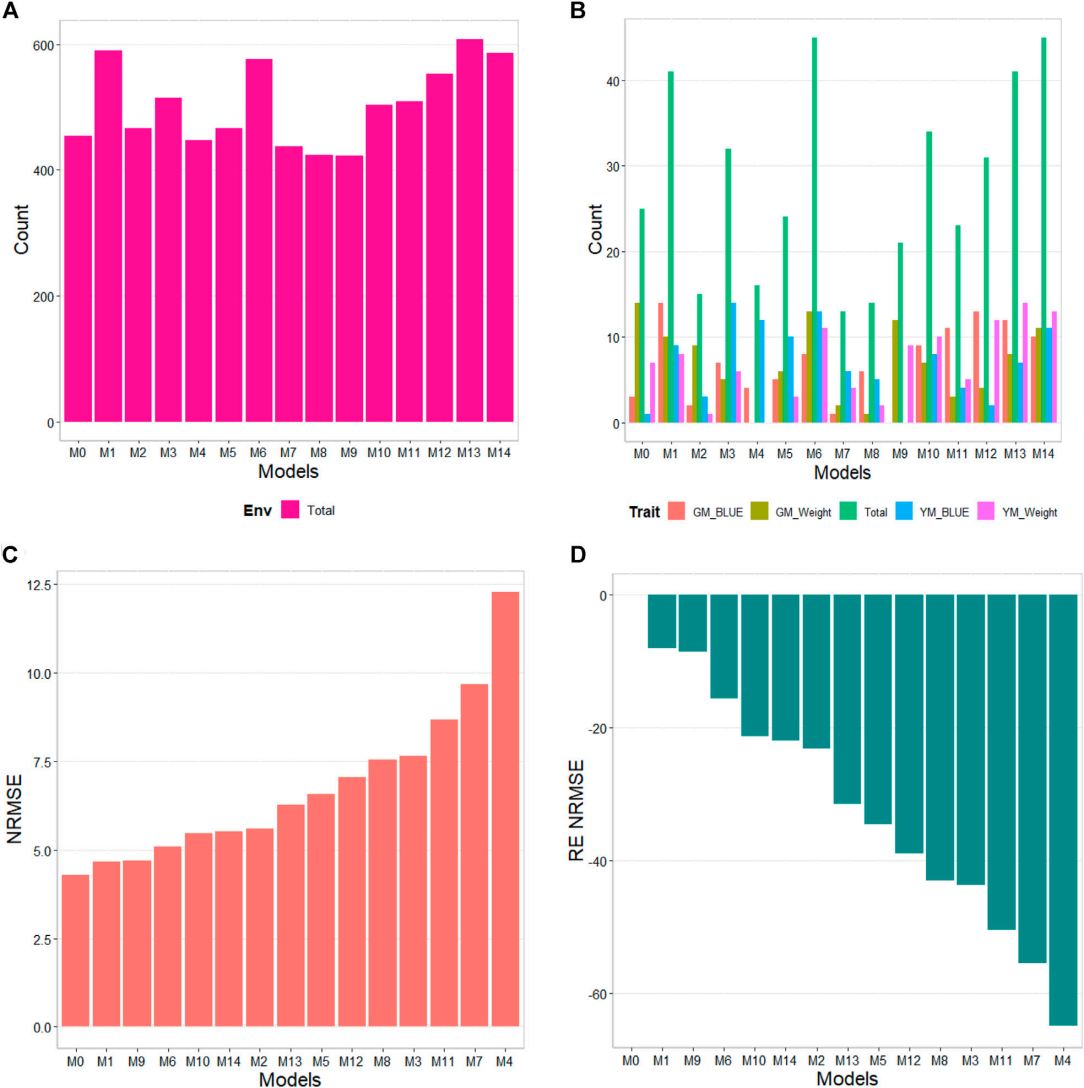
G2F_2014 data. **(A)** Count of the number of times one model is better than another, by environment. **(B)** Count of the number of times one model is better than another, by trait. **(C)** Prediction accuracy of each predictor (M0 to M14) in terms of Normalized Root Mean Squared Error (NRMSE). **(D)** Relative efficiency (RE) of each model compared to model M0, the model without environmental information.

The results of this dataset are presented in Figure 4 (see Table 6). In the count by environments, model M13 (608/1440) turns out to be the best, with a count of 608 times better than the different models of a total combination of 1440. The second-best model is M1 (590/1440), while the worst models are M9, M8, and M7 (with a count of 422/1440, 424/1440, and 438/1440, respectively). Thus, a marked difference between the best and worst models can be observed, with model M0 being the fifth-worst model.

**TABLE 6:** G2F_2014 data. Count of the number of times a model was better than another in terms of Normalized Root Mean Squared Error (NRMSE), both by environments and by traits. Prediction accuracy in terms of NRMSE. b0 denotes the absolute values of the intercept and b denotes the absolute value of 1-slope. Under an ideal model both b0 and b should be equal to zero. Relative efficiency (RE) or each model in percentage was computed regarding model M0 without environmental covariates. When the percentage is positive there is a gain in prediction accuracy regarding M0, while when the percentage is negative there is a loss in terms of prediction accuracy of any model regarding M0.

Model	Env		Trait		NRMSE	NRMSE	Cor	Cor	b0	b0	b	b
	Won	%	Won	%		RE (%)		RE (%)		RE (%)		RE (%)
	Models		Models									
M0	454	31.53	25	31.25	4.297	0.00	0.379	0.00	0.053	0.00	0.048	0.00
M1	590	40.97	41	51.25	4.675	−8.08	0.381	0.66	0.746	−92.83	0.014	247.99
M2	466	32.36	15	18.75	5.587	−23.08	0.374	−1.26	1.524	−96.50	0.307	−84.47
M3	515	35.76	32	40.00	7.632	−43.70	0.379	0.00	0.371	−85.61	0.039	23.51
M4	447	31.04	16	20.00	12.259	−64.95	0.332	−12.27	2.588	−97.94	0.392	−87.83
M5	467	32.43	24	30.00	6.569	−34.58	0.377	−0.46	0.392	−86.37	0.045	6.18
M6	576	40.00	45	56.25	5.088	−15.54	0.376	−0.73	0.002	2,126.04	0.042	14.19
M7	438	30.42	13	16.25	9.663	−55.53	0.332	−12.32	2.212	−97.58	0.393	−87.86
M8	424	29.44	14	17.50	7.543	−43.03	0.337	−10.91	2.329	−97.71	0.391	−87.80
M9	422	29.31	21	26.25	4.699	−8.56	0.293	−22.53	0.627	−91.48	0.334	−85.74
M10	504	35.00	34	42.50	5.464	−21.35	0.265	−29.91	8.143	−99.34	0.945	−94.96
M11	509	35.35	23	28.75	8.663	−50.39	0.358	−5.36	2.182	−97.55	0.306	−84.44
M12	553	38.40	31	38.75	7.035	−38.92	0.368	−2.92	1.416	−96.23	0.267	−82.11
M13	608	42.22	41	51.25	6.274	−31.51	0.378	−0.08	0.230	−76.78	0.038	25.46
M14	586	40.69	45	56.25	5.510	−22.02	0.378	−0.11	0.052	1.76	0.042	13.92

By trait, models M14 and M6 (45/80) are observed as the best models with a count of 45 models won out of 80 possible combinations. Models M13 and M1 (41/80) are positioned as the second-best models. Meanwhile, the worst model turns out to be M7 (13/80), and the second-worst model is M8 (14/80). Thus, the results by environments and traits consistently point to models M13 and M14 as the best and models M7 and M8 as the models with the poorest prediction.

In terms of NRMSE, model M0 is placed as the best of all with a NRMSE = 4.30. The second-best model is M1 (NRMSE = 4.67). Meanwhile, the worst model is M4 (NRMSE = 12.26), and the second and third-worst models are M7 (NRMSE = 9.66) and M11 (NRMSE = 8.66). Regarding Cor only model M1 was slightly better than model M0 by 0.66%, but this model was not better than model M0 in terms of intercept and slope. In this data set we observed that the environmental covariates were not significantly related to the response variable and for this reason we can observe that adding as input the environmental covariates under these circumstances is not beneficial. See details in Table 6.

### G2F_2015 data

**FIGURE 5:**
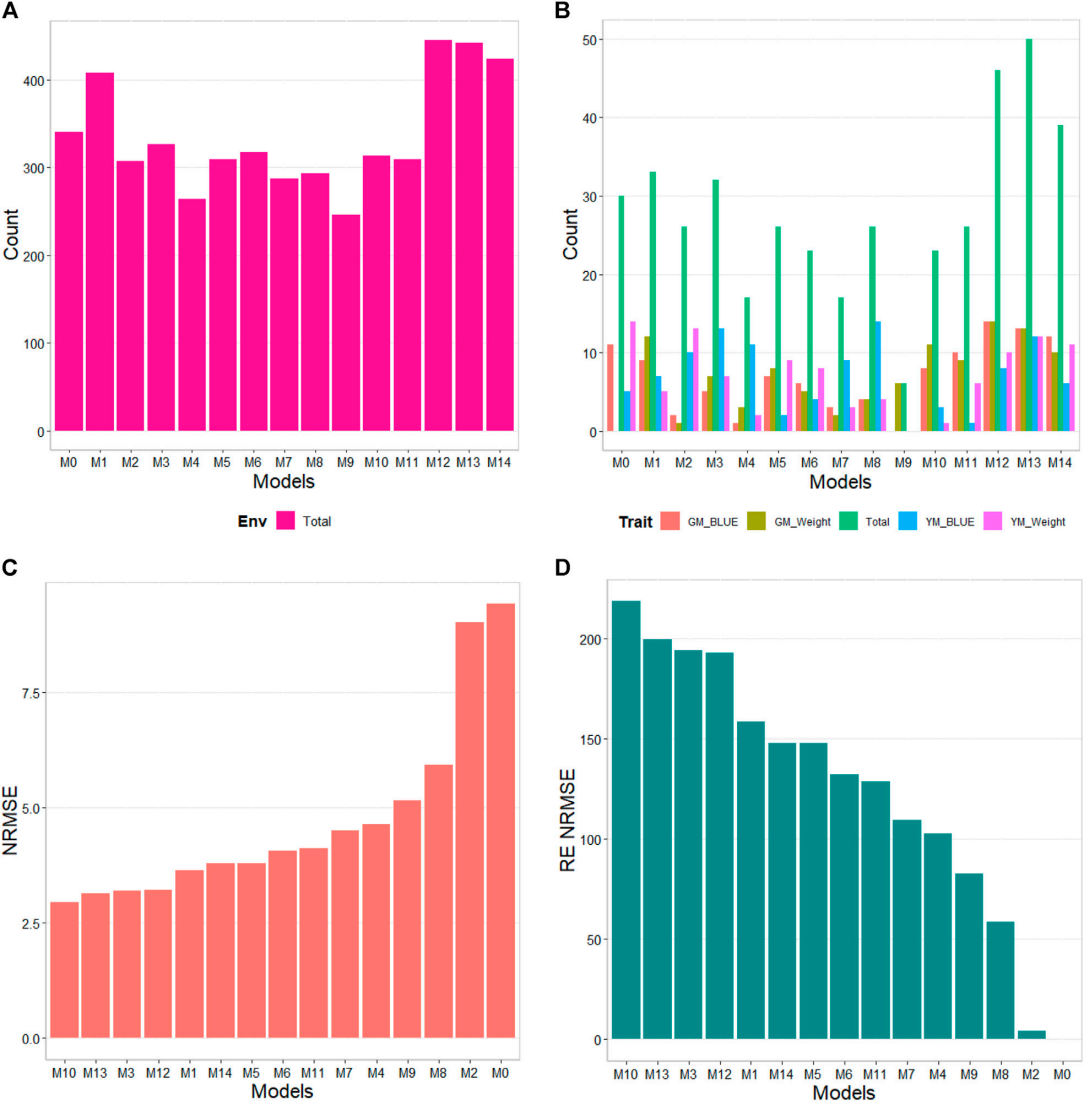
G2F_2015 data. **(A)** Count of the number of times one model performs better than another, by environment. **(B)** Count of the number of times one model performs better than another, by trait. **(C)** Prediction accuracy of each predictor (M0 to M14) in terms of Normalized Root Mean Squared Error (NRMSE). **(D)** Relative efficiency (RE) of each model compared to model M0, the model without environmental information.

The results of this dataset are presented in Figure 5 with details Table 7. In the counting by environments, model M12 (445/720) is the best, with a count of 445 times better than the different models in a total combination of 720, the second-best model is M13 (442/720), while the worst models are M9 and M4 (with a count of 246/720 and 264/720 respectively). Therefore, it is possible to observe a marked difference between the best and worst models. By trait, model M13 (50/80) is observed as the best model with a count of 50 out of 80 possible combinations. Model M12 (46/80) is positioned as the second-best model, followed by M14 (39/80). Meanwhile, the worst model is M9 (6/80), and the second-worst model was M4. Thus, the results by environments and traits are consistent in pointing to models M12 and M13 as the best and M9 as the model with the poorest prediction.

**TABLE 7:** G2F_2015 data. Count of the number of times a model was better than another in terms of Normalized Root Mean Squared Error (NRMSE), both by environments and by traits. Prediction accuracy in terms of NRMSE. b0 denotes the absolute values of the intercept and b denotes the absolute value of 1-slope. Under an ideal model both b0 and b should be equal to zero. Relative efficiency (RE) or each model in percentage was computed regarding model M0 without environmental covariates. When the percentage is positive there is a gain in prediction accuracy regarding M0, while when the percentage is negative there is a loss in terms of prediction accuracy of any model regarding M0.

Model	Env		Trait		NRMSE	NRMSE	Cor	Cor	b0	b0	b	b
	Won	%	Won	%		RE (%)		RE (%)		RE (%)		RE (%)
	Models		Models									
M0	340	35.42	30	37.50	9.411	0.00	0.523	0.00	0.336	0.00	0.090	0.00
M1	408	42.50	33	41.25	3.641	158.46	0.524	0.21	1.461	−76.97	0.098	−8.39
M2	307	31.98	26	32.50	9.015	4.39	0.480	−8.19	1.172	−71.30	0.050	79.46
M3	326	33.96	32	40.00	3.199	194.19	0.523	0.06	0.974	−65.47	0.092	−2.16
M4	264	27.50	17	21.25	4.643	102.67	0.443	−15.28	0.914	−63.18	0.166	−46.04
M5	309	32.19	26	32.50	3.794	148.04	0.523	0.10	0.788	−57.32	0.098	−8.76
M6	317	33.02	23	28.75	4.051	132.33	0.523	0.10	0.679	−50.48	0.092	−2.24
M7	287	29.90	17	21.25	4.492	109.48	0.442	−15.41	1.038	−67.59	0.167	−46.26
M8	293	30.52	26	32.50	5.929	58.73	0.439	−16.00	0.974	−65.47	0.166	−46.09
M9	246	25.63	6	7.50	5.145	82.90	0.473	−9.48	2.157	−84.40	0.070	28.20
M10	313	32.60	23	28.75	2.953	218.71	0.437	−16.46	0.964	−65.11	0.052	72.63
M11	309	32.19	26	32.50	4.114	128.75	0.475	−9.08	0.815	−58.72	0.164	−45.25
M12	445	46.35	46	57.50	3.213	192.86	0.486	−6.97	0.395	−14.86	0.134	−33.06
M13	442	46.04	50	62.50	3.139	199.82	0.522	−0.12	1.915	−82.43	0.094	−4.53
M14	424	44.17	39	48.75	3.793	148.10	0.522	−0.21	1.001	−66.37	0.084	6.35

Regarding NRMSE, model M10 is ranked as the best of all with a NRMSE = 2.95, the second-best model is M13 (NRMSE = 3.14). Meanwhile, the worst model is M0 (NRMSE = 9.41), and the second-worst model was M2 (NRMSE = 9.01). In terms of RE, compared to the worst model (M0), the gains obtained are up to 218.71% for the best model M10 and 199.81% for model M13. Also, it is noteworthy the best model, M10, was better by 23.38% compared to model M1, which is the model that takes into account all the environmental covariates without variable selection. In terms of Cor model M1 was the best with a gain of only 0.21% regarding M0, however this model was worst than model M0 in terms of **b0** and **b**, by 76.97% and 8.39%, respectively. While model M10 that was the best in terms of NRMSE was worst than model M0 in terms of Cor and **b0** by 16.46% and 65.11% respectively, however this model was better than M0 in terms of **b** by 72.63% (Table 7).

### G2F_2016 data

**FIGURE 6:**
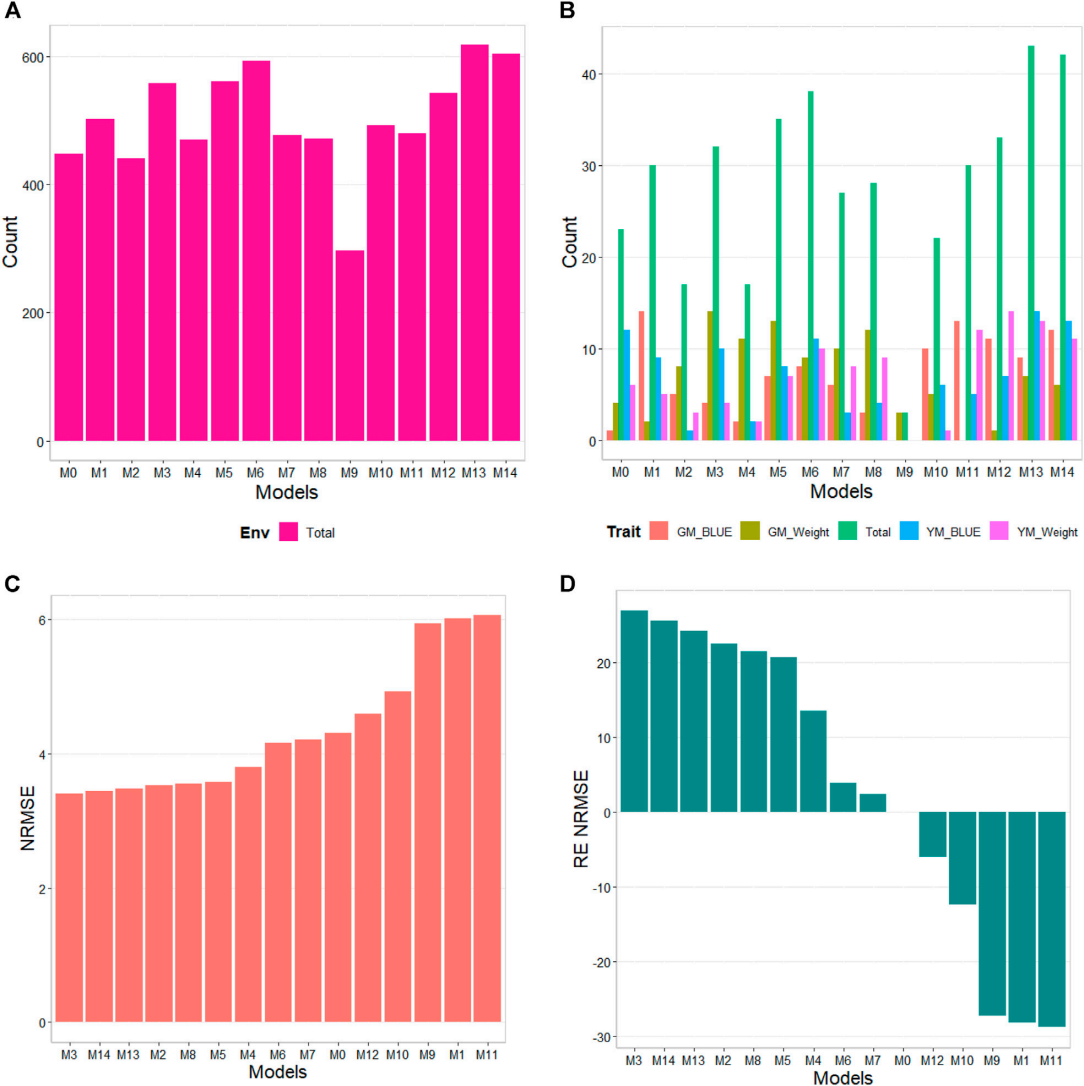
G2F_2016 data. **(A)** Count of the number of times a model is better than another, by environment. **(B)** Count of the number of times a model is better than another, by trait. **(C)** Prediction accuracy of each predictor (M0 to M14) in terms of Normalized Root Mean Squared Error (NRMSE). **(D)** Relative efficiency (RE) of each model compared to model M0, the model without environmental information.

The results of this dataset are presented in Figure 6 (Table 8). In the count by environments, model M13 (618/1440) is the best, with a count of 618 times in which it is better than the different models of a total combination of 1440. The second-best model is M14 (604/1440), while the worst models are M9 and M2 (with a count of 297/1440 and 440/1440, respectively). By trait, model M9 is observed as the best model with a count of 43 wins out of 80 possible combinations. Model M14 (42/80) is positioned as the second-best model. Meanwhile, the worst model turns out to be M9 (3/80), and the second-worst model is M2 (17/80). The results by environments and traits are consistent in pointing out model M13 as the best model and M9 as the model with the poorest prediction.

**TABLE 8:** G2F_2016 data. Count of the number of times a model was better than another in terms of Normalized Root Mean Squared Error (NRMSE), both by environments and by traits. Prediction accuracy in terms of NRMSE. b0 denotes the absolute values of the intercept and b denotes the absolute value of 1-slope. Under an ideal model both b0 and b should be equal to zero. Relative efficiency (RE) or each model in percentage was computed regarding model M0 without environmental covariates. When the percentage is positive there is a gain in prediction accuracy regarding M0, while when the percentage is negative there is a loss in terms of prediction accuracy of any model regarding M0.

Model	Env		Trait		NRMSE	NRMSE	Cor	Cor	b0	b0	b	b
	Won	%	Won	%		RE (%)		RE (%)		RE (%)		RE (%)
	Models		Models									
M0	447	31.04	23	28.75	4.312	0.00	0.493	0.00	0.479	0.00	0.089	0.00
M1	502	34.86	30	37.50	6.009	−28.24	0.491	−0.34	0.088	444.83	0.098	−9.21
M2	440	30.56	17	21.25	3.522	22.44	0.471	−4.45	1.278	−62.48	0.011	725.99
M3	558	38.75	32	40.00	3.399	26.85	0.492	−0.22	0.157	205.09	0.101	−11.68
M4	470	32.64	17	21.25	3.799	13.51	0.400	−18.78	0.880	−45.50	0.119	−25.45
M5	560	38.89	35	43.75	3.573	20.70	0.492	−0.26	0.381	25.98	0.093	−4.61
M6	593	41.18	38	47.50	4.154	3.81	0.492	−0.13	0.084	467.90	0.104	−14.20
M7	477	33.13	27	33.75	4.211	2.39	0.401	−18.73	0.889	−46.07	0.118	−24.74
M8	472	32.78	28	35.00	3.552	21.38	0.401	−18.69	0.796	−39.78	0.113	−21.29
M9	297	20.63	3	3.75	5.933	−27.32	0.430	−12.69	0.667	−28.09	0.003	2,916.95
M10	492	34.17	22	27.50	4.925	−12.45	0.406	−17.54	0.624	−23.17	0.151	−40.99
M11	479	33.26	30	37.50	6.058	−28.82	0.444	−9.87	1.014	−52.72	0.041	116.41
M12	542	37.64	33	41.25	4.589	−6.03	0.457	−7.39	1.381	−65.29	0.005	1,645.10
M13	618	42.92	43	53.75	3.474	24.13	0.492	−0.18	0.604	−20.59	0.093	−4.79
M14	604	41.94	42	52.50	3.436	25.50	0.492	−0.20	0.565	−15.12	0.092	−3.58

Regarding NRMSE, model M3 is situated as the best of all with a NRMSE = 3.40. The second-best model is M14 (NRMSE = 3.44). Meanwhile, the worst model is M11 (NRMSE = 6.06), and the second-worst model was M1 (NRMSE = 6.01). In terms of gain measured through RE, of model M3 with respect to M0, this was of 26.85%. While the best model, M3, outperformed model M1, which uses all the environmental covariates without variable selection, by 76.76%, meaning M1 decreases prediction accuracy by including all the environmental covariates in place of improving since model M0, with no environmental covariates, performed significantly better. It is important to point out that model M3 in terms of Cor and **b0** was worse than model M0 by 0.22% and 11.68% respectively, but model M3 was better than M0 by 205.09% in terms of **b**. Also, it is important to point out that any of the models M1 to M14 outperformed, in terms of Cor, the model without environmental covariates (M0). See details in Table 8.

## Discussions

Many statistical machine learning algorithms struggle to produce accurate predictions in many datasets in the context of genomic prediction because the relationship between phenotype and genotypic information and other inputs is considerably intricate and complex. But even with these difficulties, statistical machine learning methods have been adopted for genomic prediction and are helping to solve challenging problems within genetics and genomics (Ramon et al., 2020; Montesinos-López et al., 2022).

However, care needs to be taken in the application of statistical machine learning methods since if the inputs are not related to the response variable, and we use these inputs in training our prediction models we are adding noise in the modeling process. For this reason, feature selection is of paramount importance because it helps identify the most relevant and informative features from a given dataset. The goal of feature selection is to reduce the dimensionality of the data by selecting a subset of features that can provide the most accurate predictions while minimizing the risk of overfitting. Empirical evidence supports feature selection is important because it: 1) improves model performance by selecting the most relevant features, so the model can be trained more efficiently, leading to improved accuracy, and reduced error rates; 2) reduces the risk of overfitting by selecting only the most important features; 3) improves interpretability since by selecting the most important features, the model becomes more interpretable and understandable, which helps to better illustrate the underlying relationships between features and the outcome; 4) reduces data collection and storage costs since collecting and storing large amounts of data can be costly. Overall, feature selection is a crucial step in the statistical machine learning pipeline that can help to improve model performance, reduce the risk of overfitting, improve interpretability, and reduce data collection and storage costs.

In our application within the context of GS with six real datasets, we found in four out of six datasets performing variable selection significantly improve the prediction accuracy which corroborates the empirical evidence that feature selection improves prediction accuracy. In these cases, the improvement in prediction accuracy when ignoring the environmental covariates was between 14.25% and 218.71% in terms of NRMSE, however not relevant improvement was observed in terms of Pearson´s correlation. However, in the other two datasets, instead of improving the prediction accuracy with environmental covariates, we got worse results in some scenarios. These scenarios were when all the environmental covariates were included without any variable selection. For this reason, the process of selecting the important features should be done with much care and include moderate or highly correlated inputs to guarantee an improvement in prediction accuracy.

Also, we observed the resulting models with a higher predictive capacity, differ between datasets, due to the difference in the structural composition of each dataset. But, in general we observed models with selected covariates have a higher predictive capacity in terms of NRMSE. According to Buntaran et al., 2021, the prior selection of features is advantageous for model fitting when there are many features.

Regarding the predictors (M6, M7, M8, M11, M12, M13, and M14) that exclusively incorporated a single covariate, which was computed as the mean of all the selected environmental covariates, we observed competitive predictive outcomes in five of the six datasets in terms of NRMSE. This approach offers the advantage of estimating only one parameter (beta coefficient). However, it should be noted that while computing the average covariate, we altered the direction (sign) of the covariate in relation to the response variable for those covariates that exhibited a negative correlation. This adjustment ensures a positive association for each covariate. Consequently, accurately determining the direction of the association becomes crucial in order to ensure optimal performance when utilizing this average covariate.

Also, from our research, we deduce it is very challenging to incorporate some of the environmental covariates, since those that will be included during the training process only should be computed with the training set to avoid a data leakage problem. In the context of GS, with environmental covariates we have problems of data leakage when we select inadvertently the environmental covariates using the whole data set (training and testing) in place of using only the training set and for this reason we end up with overoptimistic predictions (high prediction accuracies), but when we want to use this model for real predictions, the predictions fail because data leakage lead to overfitting, The model can accurately predict the training data but performs poorly on new data. This can cause a false sense of confidence in the model’s performance and can lead to costly errors when the model is deployed in the real world.

To avoid data leakage, it is important to carefully separate the training and test data and ensure the test data represents real-world scenarios. To guarantee reasonable predictions in the testing set, we are assuming the training and testing distributions belong to the same distribution, and in this way those covariates selected with the training information will be useful for obtaining good predictions in the testing set. However, in the two out of the six data sets we can see this assumption of the same distribution between the training and testing set is not always fulfilled, so even with the variable selection process, it is not possible to improve the prediction accuracy.

When deciding which metric to use for assessing the genomic prediction performance of different machine learning models, it is important to study and investigate the advantages and disadvantages of metrics such as NRMSE and Pearson correlation between observed and predictive values. Both metrics have their advantages and disadvantages. NRMSE is beneficial when the magnitude of model errors is important. If accurately predicting absolute values is crucial for the application, NRMSE provides a measure that considers the magnitude of errors. It penalizes larger errors more heavily and can help identify models with smaller overall deviations from the actual values. On the other hand, Pearson correlation is useful when capturing the strength and direction of the relationship between variables is critical. If the focus is on assessing the linear relationship between predicted and actual values, correlation provides a measure that quantifies the strength and direction of the association.

Correlation can also be sensitive to outliers, helping identify extreme values that deviate from the overall pattern. However, correlation may not capture complex non-linear relationships well. It should be noted, however, that this study performed genomic prediction in entire environment (or years) then larger prediction errors are expected when using NRMSE, correlations, or any other metric are expected and difficult to avoid. Further research is needed to thoroughly assess the advantages and disadvantages of metrics such as NRMSE or correlation (or any other) as the basis for assessing the prediction ability of models.

In conclusion, it is important to bear in mind that the primary objective of this study was to present a proficient and pragmatic approach for integrating environmental data into the modeling process, aiming to improve prediction accuracy through the implementation of genomic selection methodology. While our results are promising in terms of NRMSE, further empirical assessments are necessary to validate our discoveries and refine the proposed methodology since the improvement is not reflected in terms of Pearson´s correlation. This involves not only incorporating feature (variable) selection techniques but also integrating feature engineering methods to enhance the predictive capabilities of genomic prediction models.

## Conclusion

In this research using six real datasets, we proposed the use of feature selection for optimally incorporating the environmental covariates in the modelling process for training genomic prediction models. We found feature selection significantly increases the prediction accuracy in terms of normalized root mean square error regarding ignoring the environmental covariates or adding all these environmental covariates without feature selection (naïve incorporation). However, the gain in prediction accuracy is data dependent, since the value can be from 0% to 218.71% in terms of Normalized Root Mean Squared Error. The key to improving prediction accuracy is to select environmental covariates that are highly correlated with the response variable. Also, we point out that the selection of the environmental covariates should be done using only the training set to avoid leakage of information problems. Finally, we encourage other researchers to apply feature selection in genomic prediction because it is an extremely powerful tool in the context of large inputs and small observations. We are convinced feature selection can be helpful to efficiently incorporate other omics data in the genomic prediction models.

## Data Availability

The original contributions presented in the study are included in the article/Supplementary Material, further inquiries can be directed to the corresponding authors.
